# FReD: The Floral Reflectance Database — A Web Portal for Analyses of Flower Colour

**DOI:** 10.1371/journal.pone.0014287

**Published:** 2010-12-10

**Authors:** Sarah E. J. Arnold, Samia Faruq, Vincent Savolainen, Peter W. McOwan, Lars Chittka

**Affiliations:** 1 School of Biological and Chemical Sciences, Research Centre for Psychology, Queen Mary University of London, London, United Kingdom; 2 School of Electronic Engineering and Computer Science, Queen Mary University of London, London, United Kingdom; 3 Silwood Park, Imperial College London, Ascot, United Kingdom; 4 Jodrell Laboratory, Royal Botanic Gardens, Kew, Richmond, United Kingdom; Centre de Recherches sur la Cognition Animale - Centre National de la Recherche Scientifique and Université Paul Sabatier, France

## Abstract

**Background:**

Flower colour is of great importance in various fields relating to floral biology and pollinator behaviour. However, subjective human judgements of flower colour may be inaccurate and are irrelevant to the ecology and vision of the flower's pollinators. For precise, detailed information about the colours of flowers, a full reflectance spectrum for the flower of interest should be used rather than relying on such human assessments.

**Methodology/Principal Findings:**

The Floral Reflectance Database (FReD) has been developed to make an extensive collection of such data available to researchers. It is freely available at http://www.reflectance.co.uk. The database allows users to download spectral reflectance data for flower species collected from all over the world. These could, for example, be used in modelling interactions between pollinator vision and plant signals, or analyses of flower colours in various habitats. The database contains functions for calculating flower colour loci according to widely-used models of bee colour space, reflectance graphs of the spectra and an option to search for flowers with similar colours in bee colour space.

**Conclusions/Significance:**

The Floral Reflectance Database is a valuable new tool for researchers interested in the colours of flowers and their association with pollinator colour vision, containing raw spectral reflectance data for a large number of flower species.

## Introduction

**Figure 1:**
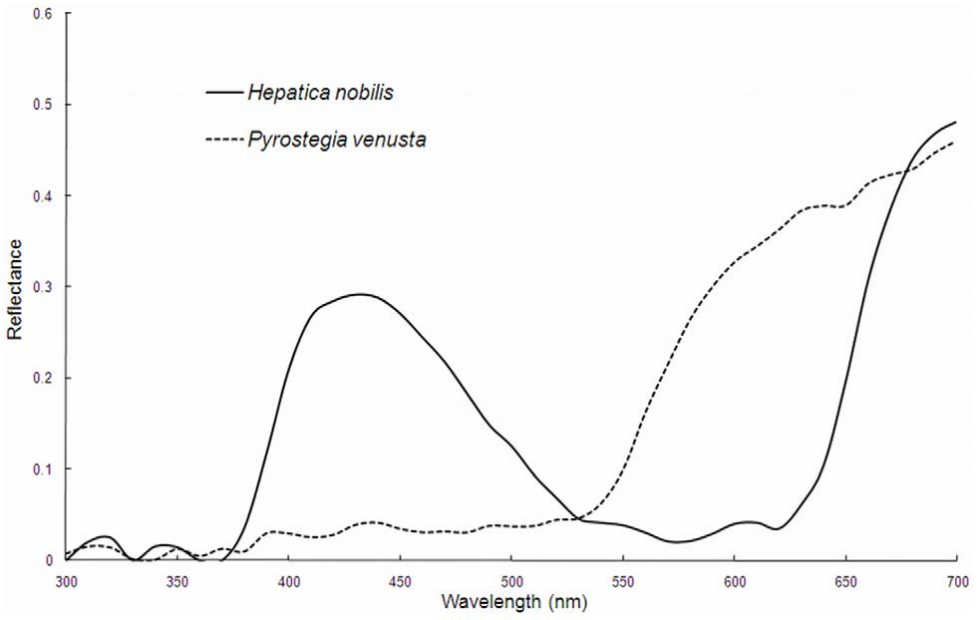
Reflectance spectra for *Hepatica nobilis* and *Pyrostegia venusta*. Samples originate from Germany and Brazil respectively. The reflectance is the proportion of light at each wavelength reflected by the sample.

Flower colour and pigmentation are of interest to researchers in areas of both developmental biology and pollination ecology [1], [2]. The colours of flowers are diverse, and have evolved under selection by their pollinators [1], [3], [4], [5]. However, flowers should not simply be categorised according to their colour appearance to a human observer, because pollinators have fundamentally different visual systems to humans (Fig.1), including sensitivity to different wavelength ranges. Insects typically have photoreceptors that respond to ultraviolet, blue and green light [6], [7]; furthermore, many insects have four or more spectral receptor types, whose sensitivity often extends into both long and very short wavelengths [8]. However, in spite of these differences, some studies investigating flower colours in plant communities have only considered these colours as humans perceive them [9], [10], [11], an oversight that has been brought up repeatedly by past scholars [12], [13], [14], [15], [16]. Such neglect of insect vision is clearly inadequate, as two colours that look distinct to a human can look similar to a pollinator, and vice versa [17], [18].

We have developed the Floral Reflectance Database (FReD) to provide free, searchable access to reflectance spectra of a large number of flowers, thus making available extensive information about flower colour that is not inherently human-biased and which can be used when considering the interactions between floral appearance and the visual systems of pollinators [19], [20]. Since the visual ecology of bees is so well understood, and they are also such important pollinators in a variety of habitats [21], the Floral Reflectance Database has devoted particular attention to modelling and predicting flower colours as they appear to bees, but it would be equally possible to analyse flower colours using another animal's visual system as the base.

To predict flower colour appearance to another animal requires us to measure the spectral reflectance of different flower parts, quantifying the proportion of light reflected by the flower at different wavelengths – including the ultraviolet [20]. This produces a reflectance spectrum (Fig. 1), and the information can be used in conjunction with a model of insect colour vision to provide information on the flower's appearance to that insect. Trichromatic bees provide one such model of colour vision: they have well-understood photoreceptor spectral sensitivity functions [6], and there is some information about their colour opponency mechanisms [22], [23]. Several colour space models have been developed [22], [24], [25]; examples include the bee colour hexagon [24], the COC (colour opponency coding) model [23] and the colour triangle [26], [27]. All have been constructed to predict the bee-subjective appearance of object colours and present this information in a graphical format. The various uses and advantages of these colour spaces are discussed in detail elsewhere [25], [28]. In colour space, a coloured stimulus occupies a single locus depending on the photoreceptor excitation signals. The distance between two loci is representative of the perceptual difference between two coloured stimuli when viewed by a bee, with more dissimilar colours positioned further apart than similar ones.

In addition to the reflectance spectra for all the samples we have reviewed, information is available in the database about their colours as perceived by a bee, including photoreceptor excitations and loci in the colour hexagon, the colour triangle and COC space. Where flowers contain parts with different colours, where possible all the flower parts have been measured and included – this is particularly relevant in light of multiple studies [29], [30], [31], [32] emphasising the importance of colour or brightness contrasts between flower parts for detection of flowers by insect pollinators, including from a distance. The database records also contain information about where each sample was collected, as well as other floral parameters and the pollinators of the respective flower species, where known. We have brought together reflectance datasets from several studies for researchers to access and use freely.

## Methods

### Data collection

The measurements in the database have been collected over the last 20 years from various sites around the world [1], [17], [33], [34]. Flower spectral reflectance functions were measured in the laboratory using a spectrophotometer. The exact technique differs somewhat between studies [1], [17], [33], [34] but all spectrophotometers work by directing an incident light on to the target object (in this case, a flower) and measuring the proportion of light reflected by the object at all wavelengths over the spectral range to which the equipment is sensitive. The flower or floral unit was always placed flat, and if the total area of flower was less than the total recording area of the spectrophotometer (0.5–1 cm^2^), several petals were carefully tiled to cover the recording area without gaps (e.g. as described in Menzel and Shmida [20]). The light source used for such measurements must contain sufficient UV as well as all human-visible wavelengths to allow accurate assessment of the flower's colour (also see [28]). In all cases, the readout was then converted into a series of reflectance measurements at wavelengths from 300 to 700 nm, in increments of 1 nm, a range that encompasses or exceeds the visible spectrum for most insects.

The database currently contains hundreds of spectral reflectance records from numerous countries, including Germany, Norway and Brazil. Where possible, the spectral reflectance functions provided are an average calculated from several identical flower parts, from multiple plants of the same species in that location, rather than simply based on a single sample.

### The Database

The Floral Reflectance Database is a MySQL (Structured Query Language – a method of coding and organising database information) database with a user interface written in PHP (Personal Home Page – a scripting language for websites). This is designed to make it easily accessible via the internet and permit users to search for samples according to specific criteria. The online release of the database functions in all major browsers and is compatible with Windows, Linux and Mac operating systems; however, users of some less common browsers may experience problems with the HexSearch (**Hex**agon **Search**) facility. The database is freely accessible for any user to search and view wavelength files.

**Figure 2:**
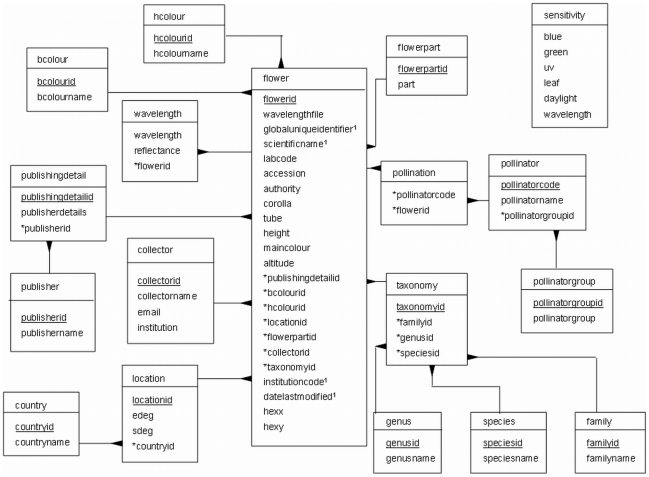
Database structure of FReD. Individual boxes indicate discrete data tables and the fields within each one. Lines linking boxes show data tables that are linked by identification codes (ID numbers); the linked fields are indicated by * in the originating table, mapping to fields that are underlined in subsidiary tables. Superscript “1” indicates those records which correspond to the mandatory DarwinCore standard fields.

The MySQL database consists of 16 tables, dealing with information on the flower sample and characteristics, location, citation information, colour, collection and taxonomy information, and the wavelength measurements themselves (Fig. 2).

- The *Flower* table is the main table, containing important details of the sample taken, including altitude (m above sea level), plant height (cm), corolla diameter (mm) and tube length (mm) measurements, colour hexagon coordinates, and if the colour information represents the dominant colour of the flower. It also contains information on the herbarium accession number of the sample, if available.
- The *Taxonomy* set of tables provide details about the species and classification of the different flower samples. Where necessary, the colour morph or subspecies of flower can be specified in the “species” field to differentiate it from other samples of the same species.
- The *Location* set of tables provide details on where the flower sample was obtained, including GPS (Global Positioning System) data where available.
- The *Flowerpart* table contains details of what flower section is being measured for each sample, e.g. calyx, tips of petals, upper lip of a zygomorphic flower, etc.
- The *Colour* tables give information on the flower colour, both as seen by a bee and a human.
- The *Pollinator* set of tables contain the information pertaining to the pollinating species, where available.
- The *Collector* table provides information about the researcher who collected the samples.
- The *Publishing* tables give information about the published source and citation information for each sample listed in the database.
- The *Wavelength* table contains the reflectance measurements themselves.
- The *Sensitivity* table is not interlinked with the flower information, but contains information on honeybee photoreceptor sensitivity, spectral components of illumination and other measurements required to calculate colour space coordinates.

The format of information contained on each flower sample in addition to the reflectance spectra is also summarised in Table 1.

**Table 1:** Summary of the searchable data fields in FReD and examples of the data format used in each.

Field	Data type	Example
Family	varchar	Fabaceae
Genus	varchar	Trifolium
Species	varchar	repens
Authority	varchar	L.
ScientificName	varchar	*Trifolium repens* L.
Collector	varchar	Chittka
Bee colour	varchar	blue-green
Human colour	varchar	white
Main flower colour	varchar	Y
Flower section	varchar	radially symmetric, whole flower upper side
Country	varchar	Norway
Town/Area	varchar	Oppdal
GPS_East	float	[longitude coordinate, where available]
GPS_South	float	[latitude coordinate, where available]
Pollinator	varchar	bumblebees, large bees
Altitude	float	900
Height	float	15
Tube length	float	3
Corolla diameter	float	15
Publication	varchar	Chittka, L. 1996 J. Theor. Biol. 181:179–196
Herbarium accession	varchar	[herbarium accession details, where available]

### Multiple samples of the same species

As previously mentioned, the database often contains multiple reflectance spectra for the same species. Different records may reflect different flower parts being sampled – e.g. the nectar guide versus the keel of the flower – in which case the part measured is specified in the “flowerpart” field. Alternatively, there may be records for different subspecies, cultivars or morphs; many species of plant have more than one floral colour morph [2]. In these cases, the “type” of plant sampled is also specified in the species field (e.g. “*Viola lutea* (w)” to indicate the white morph of *Viola lutea* (Huds.)). As the colour of the flower to human eyes is also recorder in the “human colour” field, it is possible to infer the colour morph from this information instead.

### Using the database

The database web portal consists of several user-friendly features to facilitate access to the data and provide users with additional tools for analysis and consideration of flower colours. These include:

- Search facilities
- Colour space displays
- Reflectance graphs
- Raw reflectance data downloads
- HexSearch facility, to search for flowers with similar colour hexagon loci.

### Search facilities

**Figure 3:**
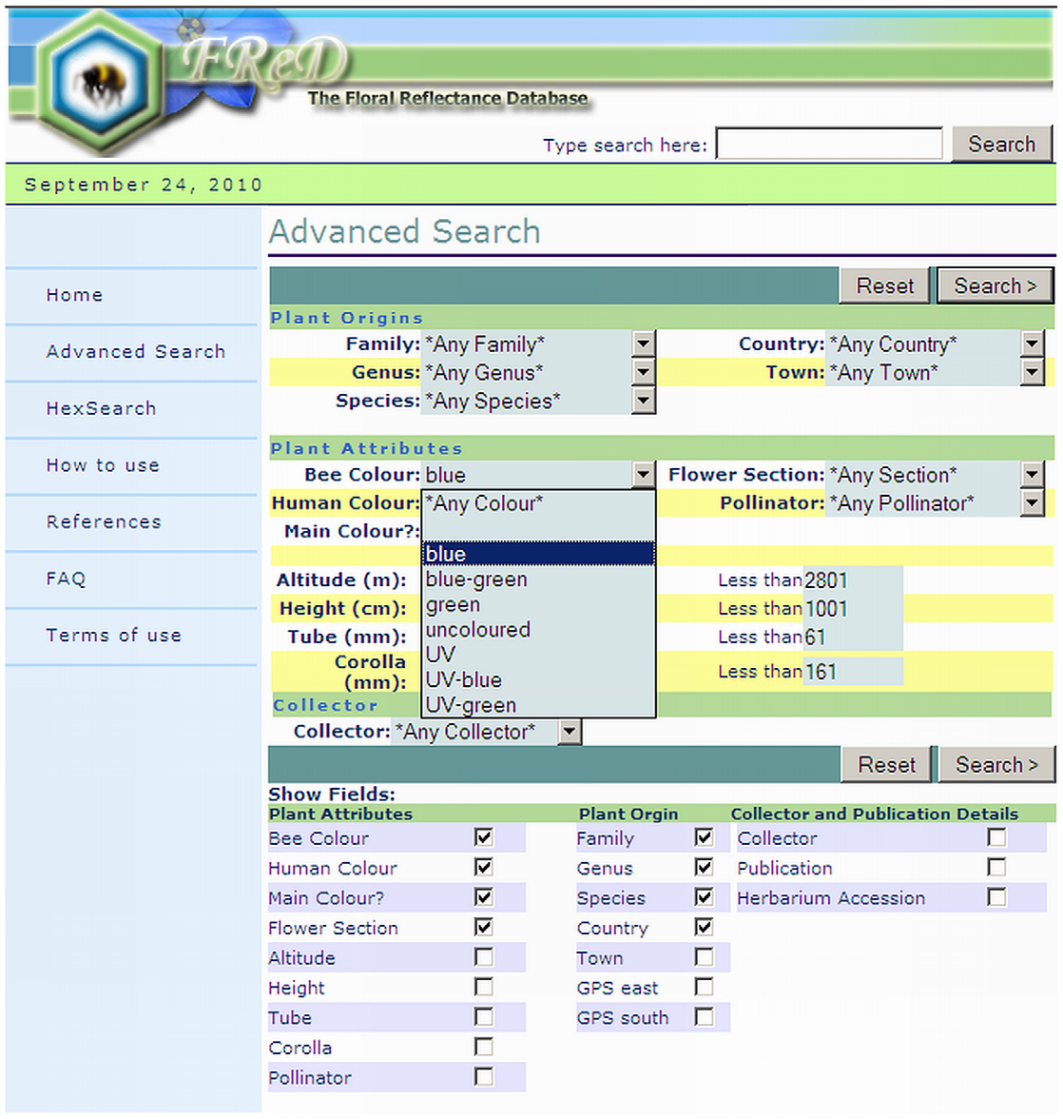
Advanced search page in FReD. This page on the FReD website allows the user to choose from many different search options in order to look for specific entries within the database, e.g. reflectance spectra for flowers from a particular genus or collected in a particular country. Most data fields are provided as drop-down menus for ease of use (here, the user is selecting “blue” from the bee colour menu); users can search for keywords or other free text using the basic search bar provided at the top of each page.

Visitors to the Floral Reflectance Database are able to use the search facilities to run basic or guided searches for flowers with specific characteristics, e.g. flowers from a particular location, of a particular species or colour, or a combination of these. The Advanced Search (Fig. 3) also allows the user to choose from which data fields he/she wishes to display results; a default selection is given, but they are free to edit this as they choose. As the basic search supports Boolean syntax (AND, OR, NOT, and use of quotes) [35], it resembles common search engines and thus is straightforward and intuitive to use.

**Figure 4:**
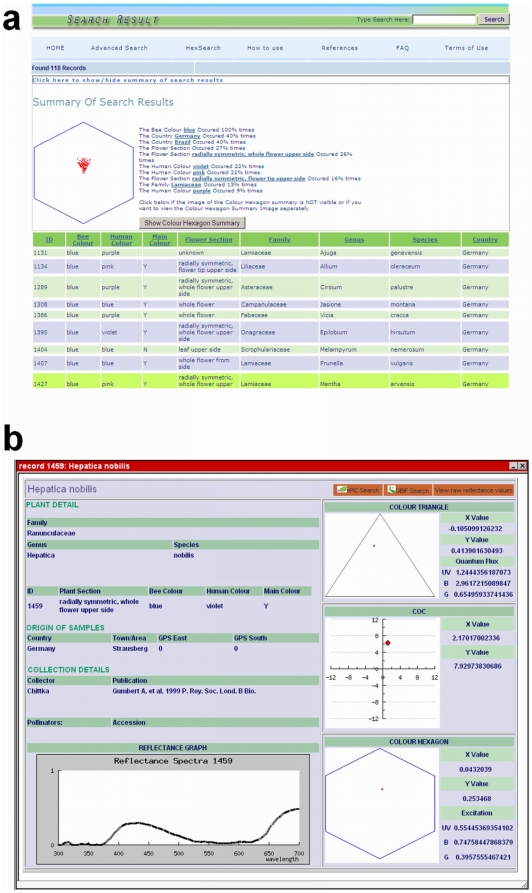
Sample search results produced by FReD in response to a search query. (The query is “blue”, looking for flowers that are either human- or bee-blue). At the top of the page of search results (a), the user has the option to display the colour hexagon (shown) and some basic descriptive statistics about the composition of the results returned. This is hidden by default to reduce page-loading times. The user can then click on an individual species record to bring up more detailed information (b) about that plant species and its floral reflectance graph, as well as viewing the colour locus for that species in three different bee colour space models.

Both types of search produce a table of results (Fig. 4a). The results can be ordered by field, by clicking on one of the column headings. A search summary is available at the top of the page (Fig. 4a), giving some descriptive statistics on the results returned (most common attributes of results, such as commonest colours, locations, etc.).

A user will then be able to view the reflectance spectra for all the search results. The use of AJAX (Asynchronous JavaScript And XML) technology keeps loading times as fast as possible by minimising the amount of unnecessary information displayed – a user is presented initially with abbreviated records, and can bring up a flower's full record in a pop-up window by clicking on an individual result (Fig. 4b). Equally, the search summary (Fig. 4a), containing a colour hexagon showing coordinates of all the results, is not displayed by default; however, it is available from a link at the top of the results page.

From the pop-up window for each flower record, there is a button to display the full reflectance data for the sample as a simple table of numeric values. From the page containing the table, it is possible to either return to the flower record, download the reflectance data in comma-separated values (.csv) format, or close the window and return to the table of search results.

### Colour space facilities

The database also has the function to display the loci of each flower on a colour hexagon diagram, a colour triangle diagram and in COC colour space. These are three different models of bee colour space [23], [24], based on the spectral sensitivities of bee photoreceptors and the colour-opponent coding mechanisms in bees [23]. Linear distances between loci within these colour spaces provide an indication of actual colour differences as they would be perceived by a bee. By making the colour loci for all three colour spaces available to users, they are able to obtain instant information about how the flower's colour might appear to a typical insect pollinator with a colour vision system similar to that of *Apis mellifera*.

The colour space coordinates are calculated taking into account the illuminating light (here, normfunction D65 [36]) and the reflectance of the background (assumed in the database to be leaves), as well as honeybee spectral sensitivities over their visible wavelength range [33]. Daylight spectral curves, leaf spectral reflectance data and honeybee spectral sensitivity curves are all taken from published literature [1], [6], [36], as are the relevant gain coefficients for the COC model [23]. Using those data, the relative excitations of the bee's three photoreceptor types can be calculated, and these three vectors can be converted into coordinates in a two-dimensional colour space diagram (e.g. the colour hexagon).

The flower records present the colour space coordinates for each sample on schematic diagrams, but also give the corresponding coordinates for each space numerically. Additionally, the excitation values for the three bee photoreceptor types are provided for users who may find these values useful. The colour space diagrams for each record are provided as Portable Network Graphics (PNG) image files that can be displayed by most modern imaging software, and can be downloaded by users if desired.

### Reflectance graph

Spectral reflectance functions for each record are displayed as a graph in the flower record, for users to assess what pattern of reflectance a flower possess, where the major reflectance peaks occur, etc. These are generated dynamically using the measurements in the Wavelength table, and displayed as a PNG file, so they can be displayed separately from the search results, and saved to a user's local hard drive if required.

### HexSearch facility

**Figure 5:**
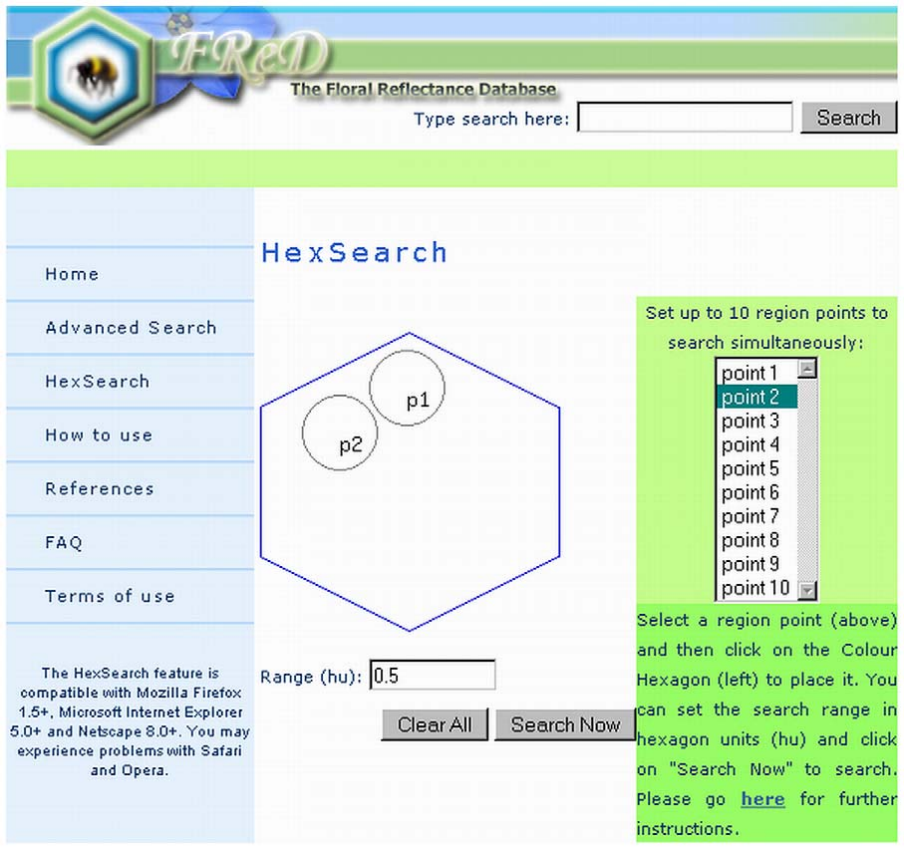
HexSearch page. The user can select up to ten points from the right-hand list, clicking to place each point at the desired location on the colour hexagon. There is an option at the bottom of the page to choose the radius of the search area for each point. When the user then selects “search now”, a page of results will be returned containing all the flower records with colour hexagon loci located within any of the search areas specified. Here, the user has selected to search two areas, each of radius 0.5 hexagon units, one area containing flowers that appear blue to bees (point 1) and one area containing flowers that appear UV-blue to bees (point 2). Therefore, the results will consist of bee-blue and UV-blue flower records only, and only those within the areas selected.

The HexSearch (Colour **Hex**agon **Search**) is an additional function, shown in Fig. 5, which may be of particular use to researchers interested in, for example, mimicry or the effects of particular pigment compounds. It permits searches for flowers with similar bee colours rather than merely searching according to gross colour category.

The user can select up to 10 loci of interest on the colour hexagon, which are searched simultaneously, and can specify their position on the hexagon by clicking in the relevant place on the map provided. The user then selects the radius of the search area (in colour hexagon units (hu)), and the function returns a page of results, comprising the flowers with colour hexagon coordinates within the area specified. Hex searches can either be general (e.g. specifying a 0.5 hu radius) or more specific (e.g. 0.05 hu). The centre point of the search can be moved as many times as required.

### Downloading reflectance data and compatibility with other databases

The database was designed to be used by researchers, and thus we are aware that users may wish to download spectral reflectance curves for their own use. This option is available by selecting the option to “view raw data” and then “view CSV file”. They can then download the reflectance measurements for each species as a.csv (comma-separated values) file, which can be imported into spreadsheets or into other databases.

In order to facilitate potential future inclusion of FReD in a larger meta-database, we have organised the database with structure in line with the international DarwinCore standard. FReD is also linked from the website of the Royal Botanic Gardens, Kew, under their lists of data and publications, in order to widen its audience to researchers who may find it useful.

In the interests of interconnectivity with other databases, all the search results returned by FReD contain links to search results for the same species in the electronic Plant Information Centre (ePIC), the large plant database run by RBG Kew, and the Global Biodiversity Information Facility (GBIF). This immediately widens the information available to users of FReD about species in the database.

## Results and Discussion

We expect the Floral Reflectance Database to be a valuable tool to researchers wishing to make between-habitat or global comparisons of floral colour; application of spectral reflectance data in studies of plant communities has already been demonstrated in multiple studies (examples: [20], [37], [38], [39]). With samples from all over the world, collected from a diverse variety of habitats, the database has applications in meta-analyses. We also anticipate its usefulness on a smaller scale, to provide detailed information on the exact colour of flowers of particular species.

By providing full reflectance spectra of all the samples, we are making available information which makes no *a priori* assumptions about the colour vision system viewing the flowers. The database provides a selection of natural, ecologically-relevant stimuli that could be used in a variety of colour modelling studies (c.f. [34], [40]). Additionally, as there are species from many plant families of differing ages, the data may, in conjunction with other information about species, have uses in studies of flower colour evolution and investigations of how floral colour relates to other characteristics.

**Figure 6:**
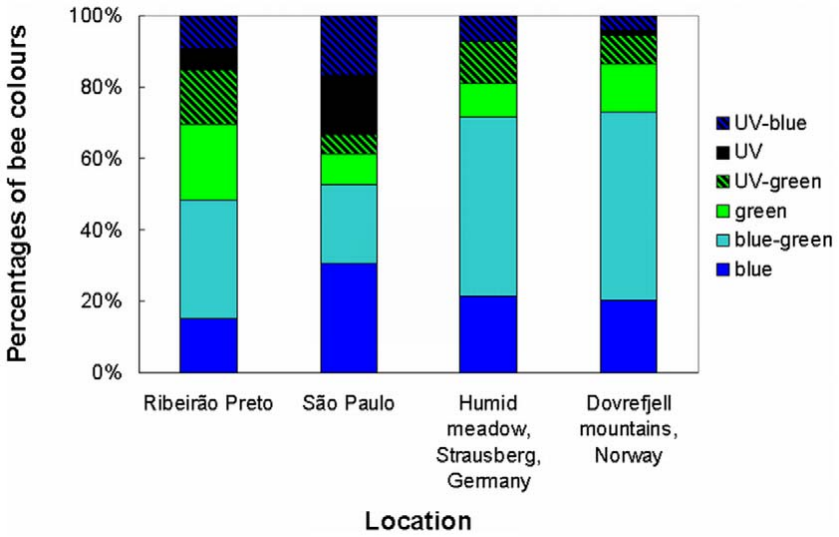
Colour compositions of flora from different worldwide locations. The graph shows the relative percentages of plant species with flowers of different bee colours in four different locations: Ribeirão Preto, Brazil; São Paulo, Brazil; Strausberg, Germany and the Dovrefjell mountains, Norway [1], [33]. The differences between the four locations are significant (χ^2^ test, χ^2^ = 42.3, *p* = 0.0002), but notably, plants with flowers of at least five out of six arbitrary bee colours are present at all locations, suggesting that in all habitats, selection is likely to result in the presence of a range of flower colours.

As an example of how the Floral Reflectance Database can be used, Fig. 6 shows the bee-colour composition of different plant communities from various parts of the world, using datasets available in FReD: two sites in Brazil (Ribeirão Preto and São Paulo) [1] – both tropical locations in South America, one from a humid meadow near Strausberg [33], [38] – a temperate location, and one from an altitudinal gradient in the Dovrefjell mountains in Norway [1], [37] – an alpine location in northern Europe. FReD provides an extensive collection of spectra from all these locations, in which all species present at each site were recorded and measured. From these spectra the bee colours can be calculated as in Chittka [24]. The figure shows that a range of colours are present at all four sites, but also that the exact percentages of different bee colours tend to differ somewhat between locations (χ^2^ test, χ^2^ = 42.3, *p* = 0.0002), principally in the proportions of blue-green-flowered species (as perceived by bees) and also UV and UV-blue flowers present. This could be due to pollinator-mediated selection with differing pressures in the differing habitats, but as previous studies have indicated that changing pollinator composition does not necessarily result in changing colour composition in a plant community [37], [38], it is also possible that the differences are due primarily to pleiotropic factors, phylogenetic constraints and/or genetic drift.

In the longer term, we intend to add more spectral reflectance readings in order to facilitate more such comparisons, and in greater detail, including data from South Africa and Costa Rica. We eventually hope to accept reflectance data from other users of the database provided that the measurements are of high quality and include the most important associated information about the sample being measured (i.e. at least species, flower section being sampled, relevant publications, location in which sample was collected). The database also has the potential to be extended to contain additional data fields of interest to pollination studies, such as details of flowering phenology.

We anticipate that as the database grows to encompass more species from diverse international locations, it will become an even more useful resource for many areas of research requiring an objective consideration of flower colours.
